# An Evolutionary Perspective of Codon Usage Pattern, Dinucleotide Composition and Codon Pair Bias in Prunus Necrotic Ringspot Virus

**DOI:** 10.3390/genes14091712

**Published:** 2023-08-28

**Authors:** Lingqi Wang, Haiting Zhao, Zhilei Wang, Shiwen Ding, Lang Qin, Runzhou Jiang, Xiaolong Deng, Zhen He, Liangjun Li

**Affiliations:** 1 College of Horticulture and Landscape Architecture, Yangzhou University, Yangzhou 225009, China; 15951142255@163.com; 2 College of Plant Protection, Yangzhou University, Yangzhou 225009, China; 19850507965@163.com (H.Z.); wangzhilei1012@163.com (Z.W.); dinshiwen1012@163.com (S.D.); qinlanguihi@163.com (L.Q.); 18994852869@163.com (R.J.); 19850508160@163.com (X.D.); 3 Joint International Research Laboratory of Agriculture and Agri-Product Safety of Ministry of Education of China, Yangzhou University, Yangzhou 225009, China

**Keywords:** prunus necrotic ringspot virus, codon usage bias, phylogenetic analysis, natural selection, dinucleotide preference, codon pair bias

## Abstract

Prunus necrotic ringspot virus (PNRSV) is a significant virus of ornamental plants and fruit trees. It is essential to study this virus due to its impact on the horticultural industry. Several studies on PNRSV diversity and phytosanitary detection technology were reported, but the content on the codon usage bias (CUB), dinucleotide preference and codon pair bias (CPB) of PNRSV is still uncertain. We performed comprehensive analyses on a dataset consisting of 359 coat protein (CP) gene sequences in PNRSV to examine the characteristics of CUB, dinucleotide composition, and CPB. The CUB analysis of PNRSV *CP* sequences showed that it was not only affected by natural selection, but also affected by mutations, and natural selection played a more significant role compared to mutations as the driving force. The dinucleotide composition analysis showed an over-expression of the CpC/GpA dinucleotides and an under-expression of the UpA/GpC dinucleotides. The dinucleotide composition of the PNRSV *CP* gene showed a weak association with the viral lineages and hosts, but a strong association with viral codon positions. Furthermore, the CPB of PNRSV *CP* gene is low and is related to dinucleotide preference and codon usage patterns. This research provides reference for future research on PNRSV genetic diversity and gene evolution mechanism.

## 1. Introduction

Prunus necrotic ringspot virus (PNRSV) belongs to the genus *Ilarvirus* of the *Bromoviridae* family [1]. The virus is spreading across the globe and it infects numerous types of stone fruits, such as plum, peach, almond, nectarine, hop, and ornamentals [2,3]. It is an important economic disease of most *Prunus* spp., encompassing both wild and cultivated species [2,4]. The virus-infected plants typically exhibit symptoms of shot-hole, rings, and chlorotic spots in leaves and bud death in trees [2]. In addition, the virus can lead to retarded fruit ripening (cherry), reduced bud-taking and declined fruit yield, resulting in significant economic losses [5]. PNRSV is a positive-sense RNA virus and has equixially symmetric polyhedron particles with diameters of 22–23 nm. The virus has a tripartite genome, which consists of positive single-strand RNA1, RNA2 and RNA3 [6]. RNA1 encode replicase proteins P1, RNA2 encode replicase proteins P2, and RNA3 encode movement protein (MP) and coat protein (CP) [7]. CP synthesis occurs via a subgenomic monocistronic mRNA (RNA 4). PNRSV can spread by grafting, or by infected seed and pollen. The primary reason behind the spread of viruses is the utilization of bud sticks obtained from plants already infected with the virus, which are then used for the further propagation of planting material.

Codons refer to three adjacent nucleotides located in messenger RNA molecules during transcription, corresponding to an amino acid, which encodes a protein polypeptide chain during translation, also known as the triplet code. In nature, in addition to the three termination codons used to synthesize termination signals, there are a total of 61 codons used to encode amino acids. Sense codons refer to the specific codons that have the capacity to encode amino acids. However, there are a total of 20 amino acids in nature [8]. Therefore, each amino acid may be encoded by multiple codons. Moreover, when different codons simultaneously encode the same amino acid, they are called synonymous codons. In the perfect condition (there is no selection pressure and neutral mutation), the nucleotide mutations at different sites of codons are often random, and the use frequencies of multiple synonymous codons that encode the same amino acid should be the same. However, in actual situations, different synonymous codons, corresponding to the same amino acid, are used at different frequencies, and they always show a tendency to use a specific synonymous codon. We call the codons with higher use frequencies optimal codons. Additionally, the phenomenon where the use frequency of synonymous codons is imbalanced is known as CUB. CUB has undergone evolutionary changes in different organisms as a result of natural selection, genetic drift mutation pressure, and this bias indicates the occurrence of adaptations aimed at evading the host’s immune system and ensuring survival [9,10,11,12,13,14]. Codon usage is commonly linked to various factors such as G+C content, gene length, secondary protein structure, compositional constraints, protein expression levels and host tRNA profiles [8,15,16,17,18].

The identification and comparison of viral metagenomes often rely on analyzing the dinucleotide composition: a genomic feature that can effectively distinguish between different viral samples [19], and the dinucleotide preference can be considered as a unique genomic “signature” for specific taxonomic groups [20,21]. Furthermore, the calculation of the dinucleotide odds ratio involves comparing the observed dinucleotide usage with the expected dinucleotide usage, and can reflect the chemistry of dinucleotides staking energies [20]. In most RNA viruses, the primary factors that induce dinucleotide preference are the avoidance of CpG and UpA dinucleotides, including *Arteriviridae*, *Bunyaviridae*, *Filoviridae*, *Flaviviridae* and *Rhabdoviridae* families [22,23,24,25]. Several reports showed that the dinucleotide preference can be influenced by CUB, mutational pressure and nucleotide composition [26].

CPB is a very stable characteristic of a species. A comprehensive analysis of CPBs derived from five vertebrates (chicken, human, pig, zebrafish, and mouse) and four arthropods (*Culex quinquefasciatus*, *Aedes aegypti*, *Ixodes scapularis*, and *Anopheles gambiae*) has conclusively demonstrated that species that are closely related exhibit a comparable CPB [27,28,29,30]. Furthermore, researchers also found a high level of correlation between CPSs from distinct mosquitoes, and deduced that CPB is a very stable characteristic of a species. It can be defined as CPB when the frequency of use of a codon pair is observably more or less than the product of its two constituent codons’ frequencies [31,32,33]. Some studies used the genomes of *Bacillus subtilis* and γ proteobacteria as models, and demonstrated that CPB is affected by tRNA–mRNA interactions in ribosomal A-site, in conjunction with the P-site third nucleotide cP3 [16,32,34]. Furthermore, CPB has a significant influence on translational elongation rates and CPB is correlated with codon bias and dinucleotide preference [16,35].

In previous research, the entire genomic sequence of cherry isolates of PNRSV in both Shanxi and Shandong provinces was identified and documented [36]. According to the statistics, there are 18 full-length genome sequences of PNRSV and 359 sequences of *CP* gene, among which the main hosts are apricot, cherry, peach, plum and rose. Countries are mainly from China, Iran, Montenegro, India, Canada and Czech Republic. Due to the major role and significant meaning of the CP protein in viruses, we selected *CP* gene sequences of PNRSV to analyze in this article. Utilizing phylogenetic analysis of the *CP* gene, PNRSV isolates from various regions across the globe are categorized into four distinct lineages, namely PV96, PV32, PE5, and CH30 [37,38,39]. However, there are relatively few synonymous codon usage patterns for PNRSV, which leads to a bias in codon usage. Dinucleotide (two consecutive nucleotides) biases and codon pair (two consecutive codons) biases were regarded as an explanation of the patterns observed in nucleotide and codon preferences [31,40]. In this study, we conducted a detailed analysis of codon usage, dinucleotide composition and codon pair usage in PNRSV based on 359 *CP* gene sequences to understand the impact of CUB, dinucleotide preference and CPB on virus evolution.

## 2. Materials and Methods

### 2.1. Virus Isolates

We downloaded all the complete *CP* sequences of PNRSV through GenBank, and we found that a total of 362 sequences can be used for the analysis of this article. The specific details pertaining to the PNRSV isolates, including information on their collection time, host origins and geographical locations, can be found in Table S1.

### 2.2. Recombination and Phylogenetic Analysis

All the PNRSV isolates in this article used CLUSTAL X2 [41] for sequence comparison and analysis, and RDP [42] software was used to perform recombination analysis on the obtained isolates using RDP, BOOTSCAN [43], GENECONV [44], SISCAN [45], MAXCHI [46], CHIMAERA [47] and 3SEQ [48]: parameter settings are software default. In order to ensure the reliability of the RDP analysis results, the *P* value obtained by each analysis method must be less than 10^−6^ and at least 5 methods have detected a recombination event before recombination has occurred. TRANSALIGN software was utilized in order to maintain the alignment of de-gapped amino acids (aa). Phylogenetic analysis of PNRSV was conducted by examining the *CP* sequences. The maximum-likelihood (ML) method, which was implemented in IQ-TREE 1.6.12, was utilized for this purpose [49]. We used the best-fit nucleotide substitution model, which was determined by the ModelFinder module in IQ-TREE 1.6.12 to construct the ML tree; and in ML tree analyses, branch support was determined by bootstrap analysis with 1,000 pseudo-replicate bootstrap values being generated. Finally, we used the iTOL software to visualize the resulting tree [50].

### 2.3. Nucleotide Composition Analysis

Nucleotide composition analyses were performed by excluding five non-bias codons, including AUG, which exclusively encodes for Methionine, and UGG, which exclusively encodes for Tryptophan, as well as three termination codons (UAA, UAG and UGA). The entire composition of nucleotides (nt) in *CP* sequences, including the proportions of A, U, G, and C, as well as the contents of AU and GC, were analyzed. Additionally, the CodonW 1.4.2 package was utilized to ascertain the nucleotide composition at the third codon locations of *CP* sequences for synonymous codons (A3s, U3s, G3s, and C3s%). Furthermore, the CAIcal SERVER (http://genomes.urv.cat/CAIcal) (accessed on 21 July 2023) [51] was utilized to determine the nucleotide composition in positions 1, 2 and 3 of codons. This analysis aimed to compute the GC content at the first site codon position (GC1), second site codon position (GC2) and third site codon position (GC3), as well as the mean of GC1 and GC2, known as GC12, for PNRSV *CP* sequences.

### 2.4. Effective Number of Codons (ENC) Analysis

ENC is commonly used as a measure to quantify the extent of codon preference within a gene. In this study, the ENC was calculated by CodonW v1.4.2 package and was employed to accurately assess the extent of CUB in the PNRSV *CP* sequences. It is represented by a numerical value that can vary between 20 and 61 [52]. The numerical value of 20 serves as an indicator of a significant level of bias, that is, the gene only uses one of each set of synonymous codons, and 61 indicates that each codon is used. When ENC > 35, it means that the gene’s CUB is weak, otherwise, it is determined that the CUB is strong.

### 2.5. Relative Synonymous Codon Usage (RSCU) Analysis

RSCU pertains to the likelihood of a specific codon being utilized in comparison to other synonymous codons that encode the corresponding amino acid [53]. The calculation method is the ratio between the frequency of a certain codon usage and its expected frequency when it is used without preference. It indicates that the codon is preferred when the RSCU > 1. In cases where the RSCU ≥ 1.5, the codon is used frequently, earning the title of a high-frequency codon. On the other hand, if the RSCU = 1, it suggests codon usage pattern has no preference.

### 2.6. Principal Component Analysis (PCA)

We performed PCA in order to ascertain the associations between different samples and a collection of interrelated variables. The data set for the *CP* sequences of each PNRSV strain was expressed as a 59-dimensional vector, where each dimension represented the RSCU value of a specific sense codon. Nevertheless, we excluded codons UGG and AUG, as well as the three termination codons. PCA analysis of PNRSV *CP* sequences was analyzed using Origin 8.0.

### 2.7. ENC-Plot Analysis

The main objective of ENC-Plot analysis is to evaluate the relationship between the ENC and GC3s. ENC-Plot analysis generates a scatter plot by representing ENC to be the dependent variable and GC3s to be the independent variable. In accordance with the concept of CUB, the construction of the standard curve is carried out under the condition that it is only affected by the mutation pressure while remaining unaffected by nature selection. If the position indicating the gene is positioned on or close to the standard curve, it suggests that the bias in codon usage is primarily affected by mutation pressure rather than selection pressure. Conversely, if the position representing the gene falls below the standard curve, it indicates that the composition of codons is predominantly affected by nature selection [54].

### 2.8. Parity Rule 2 (PR2) Analysis

We performed PR2 analysis to study the codons’ base composition. In the absence of mutation and environmental selection, the internal composition of bases within genes follows a consistent pattern that the ratio of A to T and C to G is balanced at A=T and C=G. However, owing to genetic mutations and the pressure of environmental selection, the GC usage content in genome coding sequences can vary significantly, and it is particularly obvious at the codons’ third position. This analysis method plots the calculation results of G3/(G3 + C3) and A3/(A3 + T3). The coordinate (0.5, 0.5) represents the PR2 principle (A=T, C=G).

### 2.9. Neutrality Analysis

Neutrality analysis is a valuable analytical approach aimed at quantifying the extent to which choices impact the preferential use of codons. First, determine the third GC content (GC3) and the average value of the GC content of the first and second positions (GC12) and of the gene codons, and then draw the scatter plot with GC12 and GC3 as the coordinate axes. If the *CP* gene’s points are scattered along or close to the diagonal line, which indicates a slope of 1, it signifies that the CUB is significantly impacted by mutation pressure. Conversely, the smaller slope of the curve formed by the scattered points, the preferable influence of the environmental choice will show on codon usage patterns.

### 2.10. Codon Adaptation Index (CAI) Analysis

The CAI value, which can vary between 0 and 1, is determined through the utilization of the CAIcal SERVER. This analysis aims to predict the adaptability of PNRSV to their host and a comparative analysis was conducted between multiple PNRSV isolates and their respective hosts. It was observed that isolates with higher CAIs displayed a stronger adaptability to their host organisms.

### 2.11. Dinucleotide Composition Analysis

Dinucleotides were divided into three categories on the basis of their positions in the codons. The initial two bases of a codon make up the dinucleotide_12_ motif, the last two bases make up the dinucleotide_23_ motif, and the motif at the codon–codon junctions makes up the dinucleotide_31_ motif [55]. The CodonW 1.4.2 package was used to determine the dinucleotide composition. Odds ratios represented the amount of bias in a genome for or against a particular dinucleotide in numerical form [22], and were calculated using the formula below:

ρXY = ƒXY/ƒX ƒY

where ƒX and ƒY on behalf of the frequencies of X and Y nucleotide among all four possible nucleotides A, G, C, T, and ƒXY is the frequency of the corresponding dinucleotide [56]. It showed that the dinucleotides are overrepresented when odds ratios were below 0.78, whereas the dinucleotides are underrepresented when odds ratios were above 1.23 [56].

### 2.12. Calculation of Codon Pair Bias

The natural logarithm of the ratio of the observed over anticipated occurrences of a specific codon pair is known as the codon pair score (CPS) [31]. It demonstrated that overrepresented codon pairs have positive CPS values, while statistically underrepresented codon pairs have negative CPS values. Codon pairs that are equally under- or over-represented have a CPS equidistant from 0. To calculate the CPB, which is a bias of two adjacent codons in *CP* gene coding sequences of PNRSV, we used the CPS statistics for each coding sequence as an average of the CPSs of all codon pairs present in each coding sequence.

## 3. Results

### 3.1. Recombination and Phylogenetic Analysis

Recombinant analysis of PNRSV, based on *CP* sequences by RDP, revealed three recombinants (AM491772, AM494934 and MN635763). After removing these recombinants, a total of 359 sequences could be used for subsequent analysis. For the ML tree, the best fit nucleotide substitutions model of the dataset was TIM3e+R3. Phylogenetic analyses showed all isolates are divided into four distinct groups. It also distinguished the different hosts of the isolates, and found that there was no particularly obvious correlation. Among them, cherry was the most common natural host in PV32 and PV96 lineages; in the PE5 and CH30 lineages, most isolates hosted peach (Figure S1).

### 3.2. Codon Usage Analysis

To analyze the nucleotide composition, the nucleotide content and codon usage composition of the PNRSV *CP* gene coding sequences were computed. The mean values of nucleotide were G% (28.14 ± 0.39%), U% (24.53± 0.46%), A% (24.42 ± 0.38%), and C% (22.91 ± 0.41%) for the *CP* gene. We observed that the nucleotides G and U were slightly more abundant than A and C in the PNRSV *CP* coding sequences; and the mean values of the third position’s codon composition from high to low were G3s (41.01 ± 1.04%), U3s (34.95 ± 1.13%), C3s (25.74 ± 0.91%), A3s (21.09 ± 1.36%); it is shown that the third nucleotide composition of the PNRSV *CP* gene is basically the same as that of the nucleotide composition, and G3s and U3s are also more abundant. Additionally, the mean compositions of GU% were both above the AC% for *CP* gene, respectively (52.67 ± 0.41%), (47.33 ± 0.41%). The mean values of GC12 and GC3 of the PNRSV *CP* coding sequences were (49.26 ± 0.64%) and (54.61 ± 1.30%), respectively (Table S2).

The *CP* sequences of PNRSV were divided into a dozen kinds of components according to the host by phylogenetic analysis; the top five hosts are apricot, cherry, peach, plum, and rose. The average value of their ENC is shown in Figure S2. The highest mean ENC value of 52.34 ± 0.88% is distributed for the apricot host, and the lowest mean ENC value 50.78 ± 1.74% is distributed for the rose host. The ENC > d35 of all hosts indicates that the CP protein CUB of isolates from different hosts is weak, and the structure of the coding sequence is relatively conservative.

**Figure 1:**
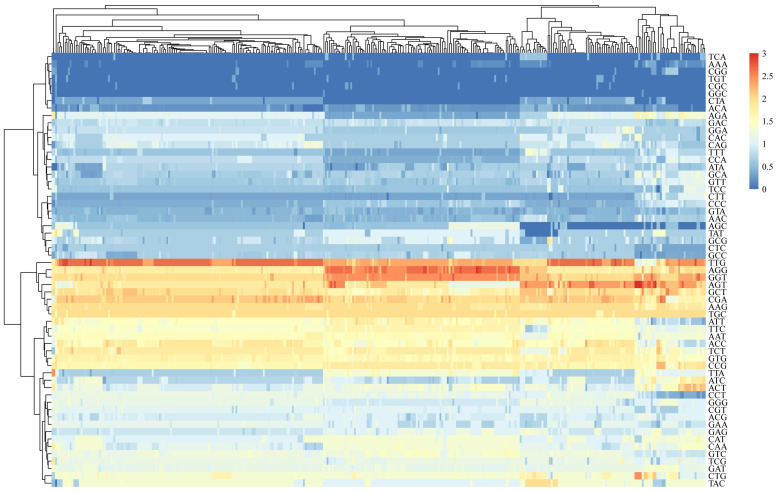
The preference of the RSCU value of 59 synonymous codons have been presented as a heatmap. As the color gets closer to red, the value of RSCU increases and the use frequency of codon gets higher. It showed that the preference of 359 *CP* sequences of PNRSV for synonymous codons is almost the same.

The RSCU value of 59 synonymous codons can be calculated intuitively, and the preference was shown directly in Figure 1. In Table S3, we found that the preferential use of codons (RSCU > 1) for the CP protein among different hosts of PNRSV is 27, and the preference of five different hosts for synonymous codons is the same. The preferred codon of the PNRSV *CP* gene ends with G/U accounting for about 70.37%. In every group, a total of twelve codons within the *CP* gene showed significant over-representation, with a mean RSCU value exceeding 1.6. Conversely, an additional fourteen codons were found to be underrepresented, with a mean RSCU value falling below 0.6. Among these preferred codons, the highest is TTG (2.42), indicating extreme overrepresentation.

**Figure 2:**
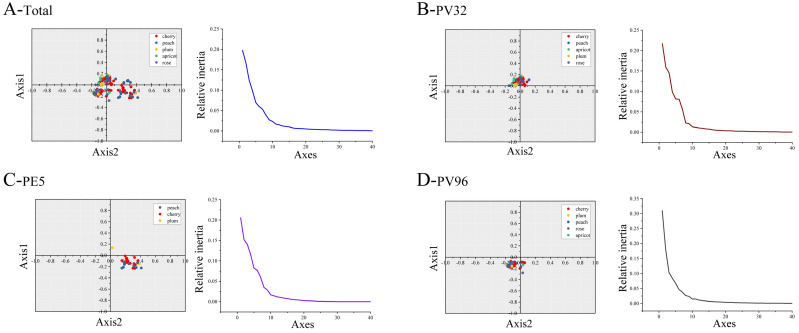
PCA of PNRSV *CP* gene based on the RSCU values of the 59 synonymous codons. (**A**) PCA in total *CP* gene of PNRSV. (**B**–**D**) PCA of PNRSV *CP* sequences in different groups. The cherry, peach, plum, apricot, rose hosts are represented in, respectively red, blue, orange, green and purple.

We performed PCA to evaluate the changes in the use of synonymous codons in PNRSV *CP* coding sequences. The initial four axes (axes 1–4) accounted for 58.60% (Figure 2), 19.76, 16.65, 12.38 and 9.81% of synonymous codon usage variation, respectively, indicating that the CUB of PNRSV *CP* gene is more affected by natural selection. At the same time, we investigated the distribution of *CP* coding sequences across various hosts by analyzing the RSCU values on the initial two axes. The PCA of the *CP* gene showed many overlapping sites between different hosts, which indicated similar codon usage trends.

**Figure 3:**
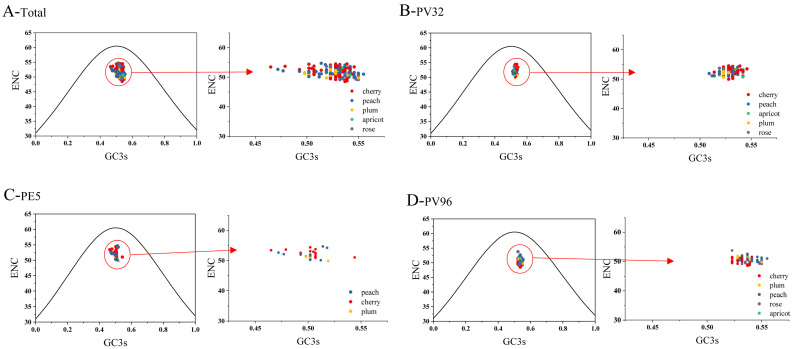
ENC-plot analysis of PNRSV *CP* gene. (**A**) ENC-plot analysis of *CP* sequences of PNRSV, with ENC against GC3s of different hosts. (**B**–**D**) ENC-plot analysis of PNRSV *CP* sequences in different groups. The cherry, peach, plum, apricot, rose hosts are represented, respectively in red, blue, orange, green and purple.

The plot generated by ENC-GC3s aims to examine the influence of mutational pressure on CUB. For both coordinates (0.5, 0.5), the center of the plot (A=T, C=G) represent no bias in nature selection or mutation pressure. Furthermore, the points locating below the standard curve indicate that its codon bias is more strongly affected by natural selection. As shown in Figure 3, all isolates of PNRSV CP protein from different hosts cluster below the expected curve, implying that natural selection exerted a more significant influence compared to mutation pressure.

**Figure 4:**
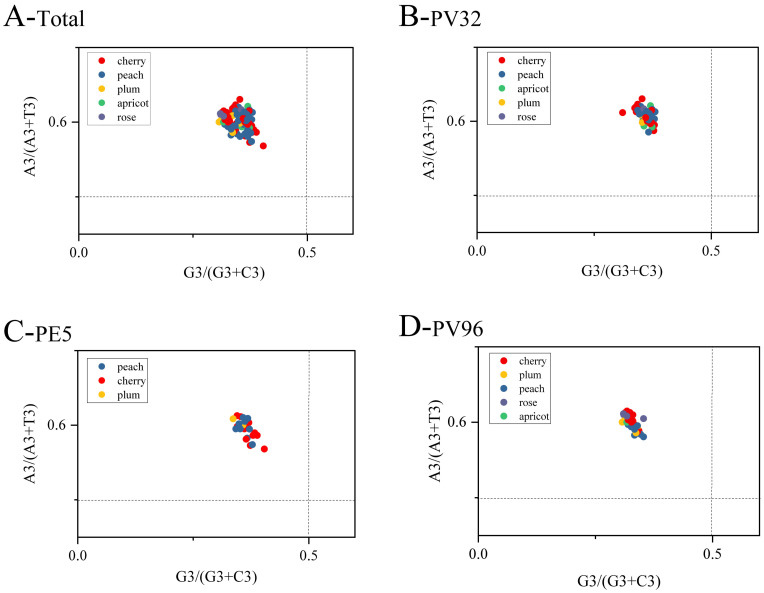
The PR2 bias plot of the PNRSV *CP* gene are shown. (**A**) PR2 analysis in total *CP* gene of PNRSV. (**B**–**D**) PR2 analysis of PNRSV *CP* sequences in different groups. The cherry, peach, plum, apricot, rose hosts are represented, respectively in red, blue, orange, green and purple.

As shown in Figure 4, the factors affecting the codon preference of the PNRSV *CP* gene were further analyzed. The PR2 bias plot takes [G/(G + C)] and [A/(A + T)] as the X and Y axes to understand the effects of natural selection and mutation on CUB of genes in both virus and host. The results show that the frequency of use of codon A is higher than that of T, and the frequency of use of codon C is higher than that of G. Among them, the third position A≠U and C≠G of all codons indicate that the codon usage of PNRSV is unequal, which indicates that the PNRSV *CP* gene codon bias is affected by both mutation pressure and natural selection.

**Figure 5:**
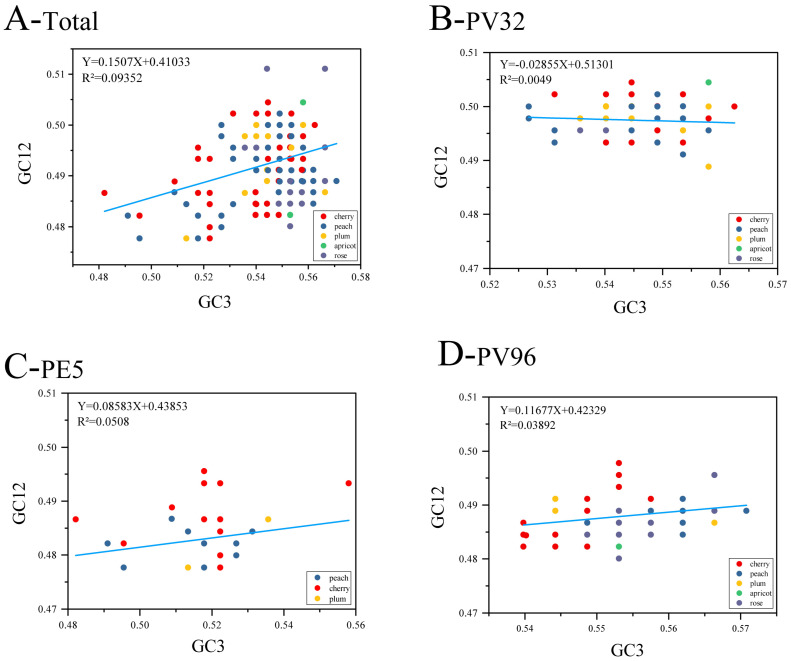
Neutrality plot analysis of PNRSV *CP* gene. (**A**) Neutrality plot analysis of GC3s against GC12s for all the PNRSV *CP* coding sequences. (**B**–**D**) Neutrality plot analysis of PNRSV *CP* sequences in different groups. The cherry, peach, plum, apricot, rose hosts are represented, respectively in red, blue, orange, green and purple.

We used GC3 and GC12 as the X and Y axes to draw a neutral graph to quantify the extent to which the CUB in *CP* sequences is impacted by mutation and natural selection. The nucleotide changes at the codons’ third position are usually considered as a mutational force, because they cannot affect the changes in amino acids. On the contrary, it can be considered as selection force if a nucleotide change results in an amino acid change. As shown in Figure 5, the linear regression coefficient (RC) of the CP protein is 0.1507 and the regression models are all statistically significant (*p* < 0.05), while the role of mutation was only 15.07% and natural selection accounts for 84.93%. Furthermore, the same trend is also observed in other groups. It is shown from the results that natural selection has a stronger effect on the CUB of the PNRSV *CP* gene than mutation pressure.

**Figure 6:**
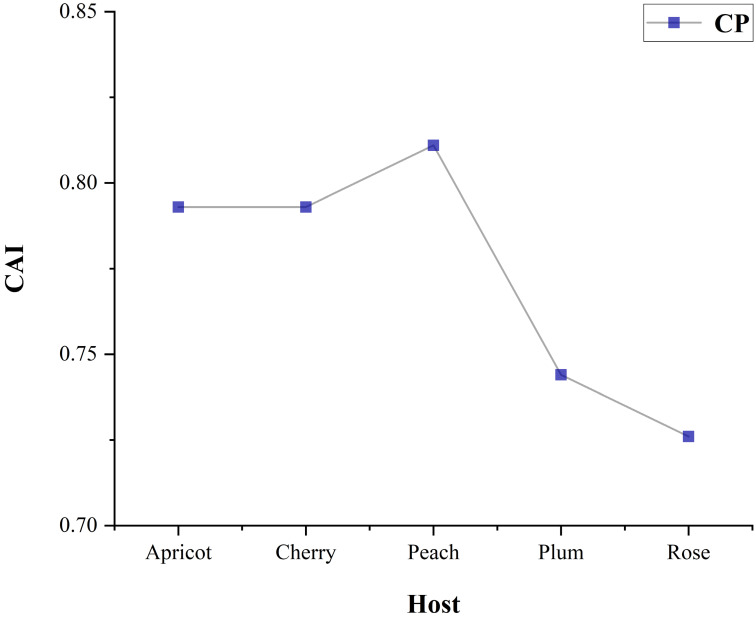
The CAI analysis of *CP* sequences of PNRSV pertaining to the native hosts. The sequences found in various hosts (apricot, cherry, peach, plum, rose) are represented on the x-axis.

The calculation of CAI values was employed to measure the extent of codon usage optimization and adaptation of the PNRSV *CP* gene towards its respective hosts. Generally, sequences possessing higher CAI values demonstrate a greater degree of adaptation to their respective hosts when compared to sequences with lower CAI values. In this particular study, the mean CAI values obtained for the *CP* gene of apricot, cherry, peach, plum, and rose were 0.792, 0.793, 0.815, 0.745, 0.728, respectively (Figure 6). These findings suggest that PNRSV have a CUB that is closer to peach than to other hosts.

### 3.3. Dinucleotide Composition Analysis

**Figure 7:**
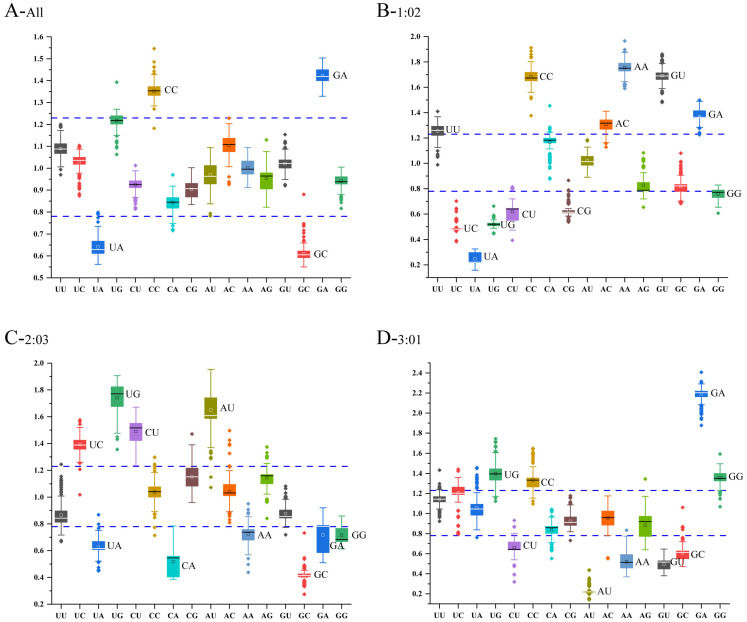
Boxplot illustrating the dinucleotide odds ratios in the PNRSV CP protein at various codon locations. (**A**) Dinucleotide Composition Analysis in total *CP* gene of PNRSV. (**B**–**D**) Dinucleotide Composition Analysis of PNRSV *CP* sequences in different groups. Odds ratios greater than 1.23 or lower than 0.78 were regarded as overrepresented or underrepresented, respectively (dotted lines). The UpU, UpC, UpA, UpG, CpU, CpA, CpG, ApU, ApC, ApA, ApG, GpU, GpC, GpA, and GpG dinucleotides are represented, respectively in black, red, blue, green, purple, yellow, aqua, brown, grass green, orange, azure, light green, gray, light red, dark blue and dark green.

We calculated the odds ratios of 16 dinucleotides for each codon position of the *CP* genome coding sequences of PNRSV (Table S4), and we estimated over- or under-representation of the 16 dinucleotides, according to the Karlin and Mrazek standard. Figure 7 shows that the dinucleotides CpC and GpA were overrepresented at all and 1-2 and 3-1 codon positions, while UpA were underrepresented at all and 1-2 and 2-3 codon positions, and GpC were underrepresented at all and 2-3 and 3-1 codon positions. We also analyzed the odds ratios of 16 dinucleotides in different groups and hosts in *CP* coding sequences of PNRSV, and found that their dinucleotide preference corresponds to the total dinucleotide preference (Figures S3 and S4).

**Figure 8:**
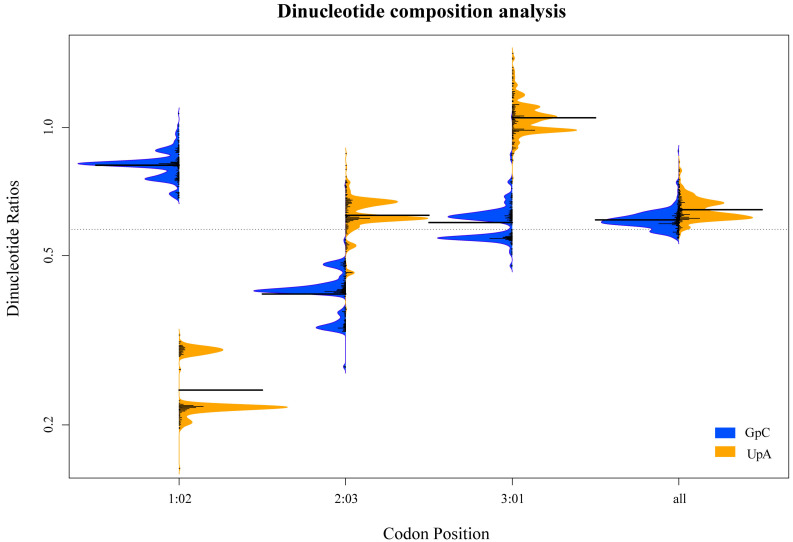
The relationship between the underrepresented dinucleotides UpA and GpC in different codon positions of PNRSV CP protein showed by “beanplot” in R.

We also used “bean plot” package in R to present the relationship between the underrepresented dinucleotides UpA and GpC, and we found that the frequency of dinucleotides UpA and GpC in different groups and hosts showed similarities (Figures S5 and S6), but it showed a clear distinction in different codon positions (Figure 8). Above all, the dinucleotide composition of the PNRSV *CP* gene showed a weak association with the viral lineages and hosts, but a strong association with viral codon positions.

### 3.4. Codon Pair Bias Analysis

**Figure 9:**
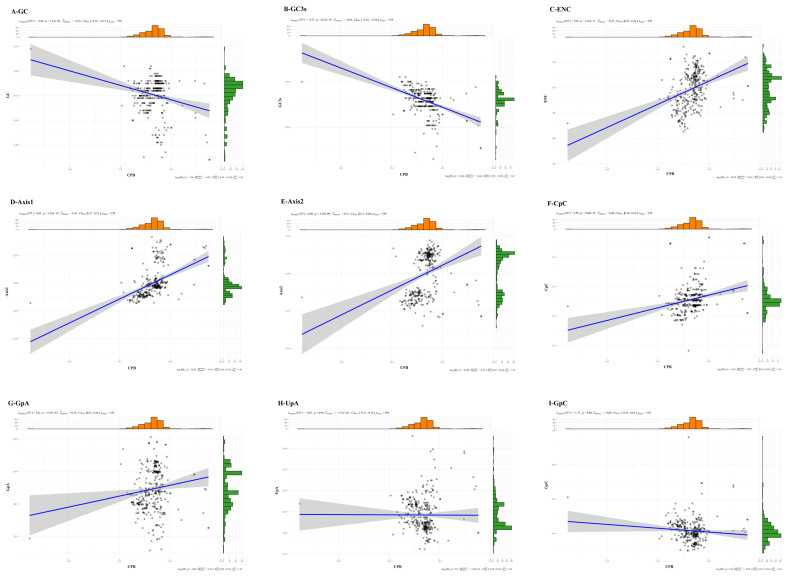
The CPB analysis in *CP* gene of PNRSV. (**A**–**I**) The correlation between CPB of PNRSV *CP* gene and the other (including GC, ENc, axis1, axis2, GC3s, UpA, CpG, CpA and UpG). [*r ˆ _Pearson* = Pearson correlation coefficient, *p* = *p* value, t(df) = *t*-test statistic for correlation coefficient with df (degrees of freedom), *n_pairs* = number of observation pairs, [*CI*] (95%) = confidence interval with 95%].

The majority of human RNA viruses have low CPBs (CPB < 0), and in this work, the CPB of the PNRSV *CP* gene sequences was also discovered to be less than 0. This finding suggests that the level of underrepresented codon pairs in PNRSV genomes is higher than the level of overrepresented codon pairs. The findings of our examination of the correlation between the CPB and the GC, ENC, Axis 1, Axis 2, and GC3s revealed that any two components were substantially associated (Figure 9A–E). Furthermore, Kunec and Osterrieder (2016) demonstrated that the primary driver of CPB is a dinucleotide preference [31]. Then, we conducted correlation analysis between CPB and the dinucleotides (CpC, GpA, UpA and GpC), and a substantial positive association was noted between CPB and the overrepresented dinucleotides GpA and CpC (Figure 9F–G), but an indistinctively negative association was noted between CPB and the underrepresented dinucleotides UpA and GpC (Figure 9H–I). In summary, the CPB of PNRSV *CP* gene is low and is related to the dinucleotide preference, codon usage patterns.

## 4. Discussion

Prunus necrotic ringspot virus (PNRSV) mainly damages stone fruit trees; it can cause significant economic losses and a high incidence rate [57]. At present, the molecular biological characteristics of the virus has been reported in many countries around the world [36,58]. According to the *CP* and *MP* gene sequences in RNA3, PNRSV isolates from different sources can be divided into four groups, such as PV96, PV32, PE5, and CH30 [37,38]. Furthermore, some articles reported that the two isolates of PchMx.Azt1 and PchMx.Azt1 were divided into one group in recent years [59]. The support rate for this grouping in this study is basically greater than 80%. The analysis results of this article show that it has no strong correlation or specificity among the different hosts in the four components. At present, there are related reports on PNRSV whole genome sequencing, genetic diversity, population structure [36,60], but there are few studies on codon usage patterns, dinucleotide preference and the CPB of the PNRSV *CP* gene. Therefore, we performed detailed analyses of PNRSV based on 359 *CP* gene sequences.

Previous studies on plant virus codons such as sugarcane mosaic virus (SCMV) [61], papaya ringspot virus (PRSV) [62], potato virus M (PVM) [63], citrus tristeza virus (CTV) [64], broad bean wilt virus 2 (BBWV2) [65], rice stripe virus (RSV) [66], rice black-streaked dwarf virus (RBSDV) [67], yellows virus (NLSYV) (NDV) [68], narcissus degeneration virus narcissus late season [68] and narcissus yellow stripe virus (NYSV) [68] have shown that the codon preference of the virus is weak, which is consistent with our analysis results. This phenomenon may be connected to the effective replication of the virus. Additionally, many factors can affect the preference of codons, including differences in base composition, natural selection factors and gene mutation factors [69,70,71]. The PR2 and GC3s, GC, and PCA analysis show that Natural selection and mutation pressure have varying degrees of influence on PNRSV. Furthermore, neutrality-plot and ENC-plot analysis found that the primary influence on the CPB of the PNRSV *CP* gene is natural selection. This is also similar to the results of many plant viruses, natural selection is dominant [61,63,64]. We guess that the transmission process may be related to changes in hosts. In contrast to viral genomes with GC-rich compositions, which typically contain codons that end with G and C, AU-rich virus genomes typically have codons ending with A and U [11,12,63]. RSCU results show that the synonymous CUB of isolates from different hosts mostly ends with G/U in the PNRSV *CP* coding sequences, similar to the RSCU observed in the Zika virus model [12]. Nucleotide composition and RSCU analysis show that the choice of preferred codons in PNRSV is mainly affected by composition restrictions (G and U), indicating the existence of mutation pressure.

Interestingly, PNRSV *CP* sequences are divided into five categories according to the hosts, and it is found that the choice of codons in different hosts means the frequency of synonymous codon usage of the isolates is the same, and the optimal codon usage pattern is also the same. It has been suggested that the choice of the best codon in viruses depends largely on their hosts. The best codon in the RSCU analysis result in different hosts is the same: both are TTG, indicating that the synonymous codon usage patterns between different hosts are almost similar, and there is no specificity between the host isolates, which may be related to the genetic relationship of the host, and they are all *Rosaceae* plants. Additionally, analysis of numerous human viruses revealed that virus coding was only marginally affected by host codon preferences [31].

To summarize, in order to obtain insight into the development of PNRSV, the *CP* gene coding sequences of PNRSV are used to analyze the precise codon usage patterns and host adaptation. The ENC-plot neutral graph analysis and neutrality-plot analysis show that natural selection is a key factor that affects the PNRSV *CP* gene codon usage pattern, but mutations also have a positive role in promoting. Therefore, studying the codon preference and evolutionary relationship of PNRSV is of great significance to its monitoring and prevention.

We discovered through dinucleotide composition analysis that UpA and GpC were significantly underrepresented in the *CP* gene of PNRSV, whereas CpC and GpA were largely overrepresented. In most single-stranded (ss) RNA viruses, the frequency of the dinucleotides UpA and CpG are suppressed [56,72]. Such dinucleotide biases clearly have a phylogenetic component. But in our study, we found that GpC replaced CpG as the underrepresented dinucleotide in the *CP* gene of PNRSV. This phenomenon may be normal, considering that PNRSV are a member of the *Bromoviridae* family, because they are different from some other single-stranded positive-sense RNA viruses in terms of size of the RNA molecules, genome segmentation and virus particle structure [73]. We also analyzed the odds ratios of 16 dinucleotides in different groups (PE5, PV32, PV96) and hosts (peach, cherry, apricot, plum, rose) in *CP* coding sequences of PNRSV and found that the dinucleotide composition of the PNRSV *CP* gene revealed a shaky relationship with the viral lineages and hosts. According to a recent study by Di Giallonardo et al. (2017), the family to which the virus belongs has a greater impact on the dinucleotide composition than that of its corresponding host in animal RNA viruses [25]. Furthermore, it showed that an increase in the frequency of UpA profoundly reduces the accumulation of plant RNA viruses and this inverse correlation between RNA accumulation and UpA frequency is applicable to mRNA-like fragments that are produced by the host RNA polymerase II, and it was confirmed by the research which used plum pox virus (PPV; *Potyviridae* family) as a model [73]. However, the biological meaning of the underrepresentation of the dinucleotide GpC in PNRSV is still unknown.

All CPB values of *CP* gene of PNRSV are below 0, indicating that underrepresented codon pairs have an advantage. The results of the correlation study showed that CUB and dinucleotide preference were highly significantly linked with the CPB of the PNRSV *CP* gene.

In this study, we cut into the CUB, host adaptability, dinucleotide preference and CPB of the PNRSV virus based on *CP* coding sequences for the first time to investigate its evolutionary changes. Our study furthers the understanding of the evolutionary changes of PNRSV, which may provide a better understanding of the origin.

## Data Availability

Tables S1–S4 in supplementary file.

## Supplementary Materials

The following supporting information can be downloaded at: https://www.mdpi.com/article/10.3390/genes14091712/s1. Figure S1: Maximum-likelihood (ML) trees calculated from the *CP* coding sequences of PNRSV; Figure S2: ENC values for the *CP* coding region sequences of PNRSV with respect to hosts (Apricot, Cherry, Peach, Plum and Rose, respectively); Figure S3: Dinucleotide composition analysis in different groups (PV32, PE5, PV96) in CP protein of PNRSV; Figure S4: The dinucleotide composition analysis of five different hosts (Apricot, Cherry, Peach, Plum, Rose) in CP protein of PNRSV; Figure S5: The relationship between the underrepresented dinucleotides UpA and GpC in different groups of PNRSV CP protein showed by “beanplot” in R; Figure S6: The relationship between the underrepresented dinucleotides UpA and GpC in different hosts of PNRSV CP protein showed by “beanplot” in R; Table S1: The details of prunus necrotic ring spot virus isolates, such as host origins, geographical locations, and collection time used in this study; Table S2: The nucleotide content and codon usage composition of PNRSV *CP* gene coding sequences; Table S3: The RSCU value of 59 codons encoding 18 amino acids according to *CP* gene sequences of PNRSV; Table S4: The dinucleotide compositions of PNRSV *CP* coding sequences.
