# Supplementary material for: An Evolutionary Perspective of Codon Usage Pattern, Dinucleotide Composition and Codon Pair Bias in Prunus Necrotic Ringspot Virus
Source: Genes (Basel). 2023 Aug 28;14(9):1712. doi: 10.3390/genes14091712 (PMC10530913; doi:10.3390/genes14091712)
Supplement: Supplementary file 1 [file genes-14-01712-s001.zip › Table S1.pdf]

Table S1. The details of prunus necrotic ring spot virus isolates, such as host origins, geographical locations, and collection time used in this study.

Accesion number	Isolate	Country	Years	Host
MW296233	EM80	Bulgaria	2018	sweet cherry
MW186680	PN11	Bulgaria	2019	sweet cherry
MT009391	PV8	Bulgaria	2018	sweet cherry
MT009388	PN5	Bulgaria	2018	sweet cherry
MT009387	PN2	Bulgaria	2018	sour cherry
MN734265	Hr2	Bulgaria	2018	sour cherry
MN635762	Vl4	Bulgaria	2019	sour cherry
MN635761	KM1	Bulgaria	2018	sour cherry
MN635760	Vl13	Bulgaria	2019	sour cherry
MN635759	St6	Bulgaria	2019	sour cherry
MN635758	Kost76	Bulgaria	2019	sweet cherry
MN635757	EM75	Bulgaria	2018	sweet cherry
MN635756	Ber56	Bulgaria	2019	sweet cherry
MF198443	Pch8	China	2015	*Prunus persica*
MH730942	P.cerasifera1	China		*Prunus cerasifera*
MH730941	Che2	China		cherry
MH730940	Che1	China		cherry
MH730939	Pea4	China		peach
MH730938	Pea1	China		peach
KF135212	ChrYT12	China: Shandong	2013	cherry
KF135211	ChrYT11	China: Shandong	2013	cherry
KF135210	ChrYT10	China: Shandong	2013	cherry
KF135209	ChrYT8	China: Shandong	2013	cherry
KF135208	ChrYT6	China: Shandong	2013	cherry
KF135207	ChrYT5	China: Shandong	2013	cherry
KF135206	ChrYT2	China: Shandong	2013	cherry
KF135205	FchrYT26	China: Shandong	2013	cherry
KF135204	AprSX3	China: Shanxi	2013	apricot
KF135203	FchrYT4	China: Shandong	2013	flowering cherry
KF135202	FchrYT3	China: Shandong	2013	flowering cherry
KF135201	PchHN9	China: Hubei	2013	peach
KF135200	PchHN2	China: Hubei	2013	peach
KF135199	PchHN1	China: Hubei	2013	peach
KF135198	PchGCS13	China: Hubei	2013	peach
KF135197	PchGCS10	China: Hubei	2013	peach
KF135196	PchGCS5	China: Hubei	2013	peach
KF135195	FchrYT5	China: Shandong	2013	flowering cherry
JX569828	417/11	Montenegro	2011	peach
JX569827	412/11	Montenegro	2011	peach

JX569826	373/11	Montenegro	2011	peach
JX569825	368/11	Montenegro	2011	peach
FJ231738	ChrT224	USA	2007	*Prunus cerasus*
FJ231737	ChrT214	USA	2007	*P. cerasus*
FJ231736	ChrT133	USA	2007	*Prunus avium*
FJ231735	ChrT77	USA	2007	*P. cerasus*
FJ231734	ChrT54	USA	2007	*P. cerasus*
FJ231733	ChrT50	USA	2007	*P. cerasus*
FJ231732	ChrT43	USA	2007	*P. avium*
FJ231731	ChrT42	USA	2007	*P. avium*
FJ231730	ChrT2	USA	2007	*P. cerasus*
HQ833200	Fpch-j	China: Zhejiang		ornamental peach
HQ833199	Pch-b	China: Hubei		peach
HQ833198	Fchr-r	China: Shandong		flower cherry
AY037791	KU	Slovakia		sour cherry
AY037790	NT	Slovakia		sour cherry
AY037789	YUG	Slovakia		plum
AY037788	GG	Slovakia		plum
ON088602	PNRSV-Ruzyne	Czech Republic	2017	*P. cerasifera*
MF069046	Cigany	Czech Republic	2016	sour cherry
MF069040	Rannaja 46	Czech Republic	2015	sour cherry
JF333586	DJ1-1	China	2010	*P. avium*
AY007217				
KC965105	HSY4-1	China: Taian, Shandong Province	2011	*P. avium*
KT444703	ChrYL	China	2015	sweet cherry
KX650619	SCh-Taian	China	2014	sweet cherry
L38823				*P. persica*
OM362557	MUSSERWC50	USA	2020	*Prunus serotina* (wild black cherry)
OM362556	MUSSERWC49	USA	2020	*P. serotina* (wild black cherry)
OM362555	MUSSERWC20	USA	2019	*P. serotina* (wild black cherry)
OM362554	MUSSERWC14	USA	2019	*P. serotina* (wild black cherry)
OM362553	MUSSERWC8	USA	2019	*P. serotina* (wild black cherry)
OM362552	MUSSERWC1	USA	2019	*P. serotina* (wild black cherry)
OM362551	WCT15	USA	2021	*P. serotina* (wild black cherry)
OM362550	WCT9	USA	2021	*P. serotina* (wild black cherry)

OM362549	28CLSH	USA	2020	*P. serotina* (wild black cherry)
OM362548	003WCMC	USA	2020	*P. serotina* (wild black cherry)
OM362547	WCA3	USA	2020	*P. serotina* (wild black cherry)
OM362546	WCA1	USA	2020	*P. serotina* (wild black cherry)
OM362545	WCGA2	USA	2021	*P. serotina* (wild black cherry)
OM362544	WCGA1	USA	2021	*P. serotina* (wild black cherry)
MW207218	PN15	Bulgaria	2019	sweet cherry
MN722634	Rad	Bulgaria	2019	sour cherry
MN722633	Nef4	Bulgaria	2018	sour cherry
MN656197	YJ5	China	2019	China rose
MN656194	YJ4	China	2019	China rose
NC004364	4/30			*P. persica*
MH282500	21716931-P	United Kingdom	2017	Morus sp.
MH282499	21716931-N	United Kingdom	2017	*Morus alba*
MF145112	SDChr10-2	China: Shandong	2016	sweet cherry
MF145111	SDChr10-1	China: Shandong	2016	sweet cherry
MF145110	SDChr96-4	China: Shandong	2016	sweet cherry
MF145109	SDChr96-3	China: Shandong	2016	sweet cherry
MF145108	SDChr96-2	China: Shandong	2016	sweet cherry
MF145107	SDChr96-1	China: Shandong	2016	sweet cherry
MF145106	SDChr104	China: Shandong	2016	sweet cherry
MF145105	SDChr93	China: Shandong	2016	sweet cherry
MF145104	SDChr62	China: Shandong	2016	sweet cherry
MF145103	SDChr73	China: Shandong	2016	sweet cherry
MF145102	SDChr64-4	China: Shandong	2016	sweet cherry
MF145101	SDChr64-3	China: Shandong	2016	sweet cherry
MF145100	SDChr64-2	China: Shandong	2016	sweet cherry
MF145099	SDChr64-1	China: Shandong	2016	sweet cherry
MF145098	SDChr66	China: Shandong	2016	sweet cherry
MF145097	SDChr11	China: Shandong	2016	sweet cherry
MF145096	SDChr15-5	China: Shandong	2016	sweet cherry
MF145095	SDChr15-1	China: Shandong	2016	sweet cherry
MF145094	SDChr15-6	China: Shandong	2016	sweet cherry
MF145093	SDChr95	China: Shandong	2016	sweet cherry
MF145092	SDChr98	China: Shandong	2016	sweet cherry
MF145091	SDChr112	China: Shandong	2016	sweet cherry
MF145090	SDChr92-18	China: Shandong	2016	sweet cherry
MF145089	SDChr97-4	China: Shandong	2016	sweet cherry

MF145088	SDChr97-1	China: Shandong	2016	sweet cherry
MF145087	SDChr79	China: Shandong	2016	sweet cherry
MF145086	SDChr117	China: Shandong	2016	sweet cherry
MF145085	SDChr8	China: Shandong	2016	sweet cherry
MF145084	SDChr5	China: Shandong	2016	sweet cherry
MF145083	SDChr2	China: Shandong	2016	sweet cherry
MF145082	SDChr130	China: Shandong	2016	sweet cherry
MF145081	SDChr131-2	China: Shandong	2016	sweet cherry
KY484024	ZK15			
KY484023	SH12			
KY484022	SH6			
KY484021	SH4			
KY484020	SH3			
KY484019	PN100			
KY484018	PN50			cherry
KY484017	PN10			almond
KY484016	PN3			almond
KY484015	PN1			peach
KY484014	P2			peach
KU977382	Cherry 13	China	2015	cherry
KU977381	Cherry 7	China	2015	cherry
KU977380	Cherry 3	China	2015	cherry
KU977379	YTL1	China	2015	*P. cerasifera*
KU977378	Plum 3	China	2015	plum
KU977377	Peach 15	China	2015	peach
KU977376	Peach 11	China	2015	peach
KU977375	Peach 8	China	2015	peach
KU977374	Peach 2	China	2015	peach
KX353935	SHN-40	Iran		nectarine
KX353934	SHN-31	Iran		nectarine
KX353933	SHN-12	Iran		nectarine
KX353932	SHN-6	Iran		nectarine
KX353931	ZK-15	Iran		nectarine
KX353930	ZKN-52	Iran		nectarine
KX574326	MA-BR	Brazil	2014	*P. cerasifera*
KX574325	DN-BR	Brazil	2014	*P. persica*
KJ958527	Rose-Br	Brazil	2014	Rosa sp.
JQ005058	Pch13	Canada	2011	peach
JQ005057	Pch11	Canada	2011	peach
JQ005056	Pch10	Canada	2011	peach
JQ005055	Pch9	Canada	2011	peach
JQ005054	Pch8	Canada	2011	peach
JQ005053	Pch7	Canada	2011	peach
JQ005052	Pch6	Canada	2011	peach

JQ005051	Pch5	Canada	2011	peach
JQ005050	Pch4	Canada	2011	peach
JQ005049	Pch3	Canada	2011	peach
JQ005048	Pch2	Canada	2011	peach
JQ005047	Pch1	Canada	2011	peach
JQ005046	Chr18	Canada	2011	cherry
JQ005045	Chr17	Canada	2011	cherry
JQ005044	Chr16	Canada	2011	cherry
JQ005043	Chr15	Canada	2011	cherry
JQ005042	Chr14	Canada	2011	cherry
JQ005041	Chr13	Canada	2011	cherry
JQ005040	Chr12	Canada	2011	cherry
JQ005039	Chr11	Canada	2011	cherry
JQ005038	Chr10	Canada	2011	cherry
JQ005037	Chr9	Canada	2011	cherry
JQ005036	Chr8	Canada	2011	cherry
JQ005035	Chr7	Canada	2011	cherry
JQ005034	Chr6	Canada	2011	cherry
JQ005033	Chr5	Canada	2011	cherry
JQ005032	Chr4	Canada	2011	cherry
JQ005031	Chr2	Canada	2011	cherry
JQ005030	Ch1	Canada	2011	cherry
JN416776	Pch12	Canada	2011	peach
JN416773	Chr3	Canada	2011	cherry
U57046	30/4			*P. persica*
AF332616	Kirka II/7			plum
AF332615	F12/1 237			cherry
AF332613	SX/2			plum
AF332618	XII/10			sour cherry
AF332617	V/11			sour cherry
AF332614	WWL I/7			plum
AF332612	dzik			cherry
AF332611				plum
AY948441	RM-5	India:Hessaraghatta, Karnataka		rose
AF013287	SW6			
AF013286	Prune			
AF013285	Mission			
FJ610344		China: Haining		rose
FJ610343		China:Xi'an		cherry
FJ610342		China:Kunming		rose
FJ610341	CHCP	China		
AY948440	RM-2	India: Chettalli, Karnataka		rose

Y07568				Malus sp.
ON667997	YJ4(R-A)	China	2022	China rose
ON667996	YJ4(R-A)	China	2022	rose
ON667995	YJ4(R-A)	China	2022	rose
ON304061	XJ07	China: Shihezi	2019	*P. persica*
ON060761	YJ3	China	2019	rose
OM650244	M2	Lebanon	2021	rose
OK625809	KK10	India	2018	rose
MZ451065	13TF169		2013	Prunus
MZ451064	13TF133		2013	Prunus
MZ451063	13TF132		2013	Prunus
MZ451062	13TF118		2013	Prunus
MZ451061	13TF106		2013	Prunus
MZ451060	13TF102		2013	Prunus
MZ451059	13C278		2013	Prunus
MZ451058	13C277		2013	Prunus
MZ451057	13C268		2013	Prunus
MZ451056	13C260		2013	Prunus
MZ451055	13C258		2013	Prunus
MZ451054	13C257		2013	Prunus
MZ451053	13C243		2013	Prunus
MZ451052	13C241		2013	Prunus
MZ451051	13C233		2013	Prunus
MZ451050	13C227A		2013	Prunus
MZ451049	13C222		2013	Prunus
MZ451048	13C221		2013	Prunus
MZ451047	13C214		2013	Prunus
MT537134	BJ-YBY	China	2019	*Rosa chinensis*
LC579924	PP11	China	2019	*P. persica*
LC431515		South Korea	2017	*P. persica*
LC382468	Acot	India	2015	*Prunus armeniaca*
KY883335	Q15R3N	Australia	2015	
KY883325	PCH4R3N	Australia	2015	
KY883322	M32R3	Australia	2015	
KY488188	Taif	Saudi Arabia: Taif	2016	*Rosa damascena*
DQ983493	I-9			apricot (*P. armeniaca*)
FJ546092	PchMx.Unk1	Mexico	2007	*P. persica*
,lFJ546091	PchMx.Azt2	Mexico	2007	*P. persica*
FJ546090	PchMx.Azt1	Mexico	2007	*P. persica*
EU368738	PNRSV-Mk	Hungary		sour cherry
EU368737	PNRSV-PL38	Italy		plum
EU368736	PNRSV-AL17	Italy		*Prunus dulcis*
EU368735	PNRSV-AL1	Australia		*P. dulcis*
EU869295		China	2008	sweet cherry

DQ983499	Emp			plum (*Prunus domestica*)
DQ983498	143			rose
DQ983497	11			rose
DQ983496	U9			plum (*P. domestica*)
DQ983495	Bl			Plum (*P.domestica*)
DQ983494	cz2			sweet cherry (*P. avium*)
DQ983492	B56			Peach (*P. persica*)
DQ983491	JW			sour cherry (*P. cerasus*)
HQ833197	Nec-g	China: Hubei		nectarine
HQ833196	Nec-f	China: Hubei		nectarine
HQ833195	Nec-e	China: Hubei		nectarine
HQ833194	Pch-a	China: Hubei		peach
HQ833193	Chr-m	China: Henan		cherry
HQ833192	Chr-q	China: Shandong	2011	cherry
HQ833191	Chr-p	China: Shandong	2011	cherry
AY684271	Yunnan			
EF565269	PchUy.jun1	Uruguay		Peach
EF565268	PchUy.ear1	Uruguay		Peach
EF565267	PlmUy.gol1	Uruguay		Plum
EF565266	PchBr.unk3	Brazil		Peach
EF565265	PchBr.unk2	Brazil		Peach
EF565264	PchBr.unk1	Brazil		Peach
EF565263	PlmCl.dag1	Chile		Plum
EF565262	PlmCl.fri1	Chile		Plum
EF565261	PlmCl.bla1	Chile		Plum
EF565260	PlmCl.mrb1	Chile		Plum
EF565259	PchCl.sum1	Chile		Peach
EF565258	PchCl.aug1	Chile		Peach
EF565257	PchCl.ric1	Chile		Peach
EF565256	PchCl.loa2	Chile		Peach
EF565255	PchCl.loa1	Chile		Peach
EF565254	PchCl.pom1	Chile		Peach
EF565253	NctCl.aug1	Chile		Nectarine
EF565252	NctCl.ear1	Chile		Nectarine
EF565251	ChrCl.roy1	Chile		Cherry
EF565250	ChrCl.swe1	Chile		Cherry
EF565249	ChrCl.bin1	Chile		Cherry
EF565248	ChrCl.cor1	Chile		Cherry
EF565247	AlmCl.car1	Chile		Almond
AJ969110	Pal	India:Himachal Pradesh, Palampur		Pelargonium
AJ969095	Rose	India:North Himalayan region		Rose
AJ619958	rose	India		

AJ133203	ChrIt.lam1	Italy		cherry
AJ133213	PlmIt.mrb1	Italy		Plum
AJ133212	PlmIt.clf1	Italy		Plum
AJ133211	PlmAl.unk1	Albania		Plum
AJ133210	ChrIt.bla1	Italy		Cherry
AJ133209	ChrIt.mrs1	Italy		Cherry
AJ133208	NctSp.mur1	Spain		nectarine
AJ133207	PchIt.mry1	Italy		Peach
AJ133206	PchTu.unk1	Tunisia		peach
AJ133205	PchIt.may1	Italy		Peach
AJ133204	AlmIt.cor1	Italy		Almond
AJ133202	AlmIt.pre1	Italy		Almon
AJ133201	AprIt.try1	Italy		Apricot
AJ133200	AprIt.nap1	Italy		Apricot
AJ133199	AprIt.caf1	Italy		apricot
OM677864	MUSSERP25	USA	2020	*P. persica*
OM677863	MUSSERP21	USA	2020	*P. persica*
OM677862	MUSSERP18	USA	2020	*P. persica*
OM677861	MUSSERP17	USA	2020	*P. persica*
OM677860	MUSSERP16	USA	2020	*P. persica*
OM677859	MUSSERP14	USA	2020	*P. persica*
OM677858	MUSSERP13	USA	2020	*P. persica*
OM677857	MUSSERP12	USA	2020	*P. persica*
OM677856	MUSSERP11	USA	2020	*P. persica*
OM677855	MUSSERP10	USA	2020	*P. persica*
OM677854	MUSSERP9	USA	2020	*P. persica*
OM677853	MUSSERP8	USA	2020	*P. persica*
OM677852	MUSSERP7	USA	2020	*P. persica*
OM677851	MUSSERP6	USA	2020	*P. persica*
OM677850	MUSSERP5	USA	2020	*P. persica*
OM677849	MUSSERP4	USA	2020	*P. persica*
OM677848	MUSSERP3	USA	2020	*P. persica*
OP357944	DSMZ PV-0962	Germany: Dossenheim		*P. cerasus*
AF170171	Valticka	Czech Republic: Valtice		peach
AF170170	Na hrbu	Czech Republic: Ceske Budejovice		*P. domestica*
AF170169	Nahnuta	Czech Republic: Ceske Budejovice		prune
AF170168	sss	Czech Republic: Ceske Budejovice		prune
AF170167	UH1	Czech Republic: Holovousy		prune

AF170166	PS 7/5a	Czech Republic: Holovousy		sour cherry
AF170165	4/8	Czech Republic: Holovousy		cherry
AF170164	7/20	Czech Republic: Holovousy		cherry
AF170163	UN	Czech Republic: Unicov		sour cherry
AF170162	PS 7/12	Czech Republic: Holovousy		sour cherry
AF170161	PS 7/11	Czech Republic: Holovousy		sour cherry
AF170160	6/54	Czech Republic: Holovousy		prune
AF170159	PS 14/22	Czech Republic: Holovousy		flowering cherry
AF170158	PS 12/16	Czech Republic: Holovousy		cherry
AF170157	21/1	Czech Republic: Holovousy		cherry
AF170156	1/13	Czech Republic: Holovousy		cherry
KU179193	SD-PNRSV	China: Shandong	2015	peach
FR773524	SK25	India:Shimla, Rohru, Kuthara	2010	*P. avium*
KJ599819	FA51_M13uni-21_PNRSV	Iran	2013	*Rosa damascena*
KJ599818	FA50-esb2	Iran	2013	*R. damascena*
KJ599817	FA15_M13uni-21_PNRSV	Iran	2013	*R. damascena*
KJ599816	FA48-esb1	Iran	2013	*R. damascena*
KJ573395	Iranian	Iran	2011	*P. dulcis*
KF420289	162/12	Montenegro	2012	*P. persica*
KF420288	152/12	Montenegro	2012	*P. persica*
KF420287	149/12	Montenegro	2012	*P. persica*
KF420286	143/12	Montenegro	2012	*P. persica*
KF420285	137/12	Montenegro	2012	*P. persica*
KF420284	134/12	Montenegro	2012	*P. persica*
KF420283	132/12	Montenegro	2012	*P. persica*
KF420282	131/12	Montenegro	2012	*P. persica*
KF420281	130/12	Montenegro	2012	*P. persica*
KF420280	126/12	Montenegro	2012	*P. persica*
DQ300178	Beijing	China		
DQ003584	I-23	Poland		rose

FN548100	JK1	India:Jammu & Kashmir	2009	*P. avium*
AM408909		India		*P. persica*
AM419814		India:Kullu		*Malus domestica*
AM931161		India:Mandi, Barot		
AM920668		India:Palampur		
AM408910		India:Kullu		*P. persica*
S78312				
