# Supplementary material for: An Evolutionary Perspective of Codon Usage Pattern, Dinucleotide Composition and Codon Pair Bias in Prunus Necrotic Ringspot Virus
Source: Genes (Basel). 2023 Aug 28;14(9):1712. doi: 10.3390/genes14091712 (PMC10530913; doi:10.3390/genes14091712)
Supplement: Supplementary file 1 [file genes-14-01712-s001.zip › Table S3.pdf]

Table S3. The relative synonymous codon usage (RSCU) value of 59 codons encoding 18 amino acids according to *CP* gene sequences of PNRSV.

**Codon**	**aa**	**PNRSV**				
		**Apricot**	**Cherry**	**Peach**	**Plum**	**Rose**
TTT	F	0.56	0.51	0.62	0.55	0.47
TTC	F	**1.44**	**1.49**	**1.38**	**1.45**	**1.53**
TTA	L	0.71	0.97	0.95	0.83	1.21
TTG	L	**2.63**	**2.39**	**2.44**	**2.55**	**2.35**
CTT	L	0.33	0.39	0.43	0.41	0.35
CTC	L	0.65	0.63	0.61	0.64	0.65
CTA	L	0.40	0.20	0.21	0.29	0.09
CTG	L	1.28	1.43	1.35	1.28	1.34
ATT	I	**1.56**	**1.48**	**1.35**	**1.41**	**1.64**
ATC	I	0.85	0.88	1.00	1.04	0.74
ATA	I	0.60	0.64	0.65	0.54	0.62
GTT	V	0.66	0.68	0.72	0.69	0.60
GTC	V	1.21	1.21	1.19	1.21	1.34
GTA	V	0.46	0.47	0.46	0.46	0.42
GTG	V	**1.67**	**1.64**	**1.63**	**1.64**	**1.64**
TCT	S	1.81	1.73	1.61	1.71	1.74
TCC	S	0.58	0.60	0.65	0.63	0.64
TCA	S	0.05	0.01	0.08	0.05	0.00
TCG	S	1.22	1.17	1.20	1.21	1.16
AGT	S	**1.70**	**1.94**	**1.96**	**1.84**	**1.76**
AGC	S	0.63	0.54	0.49	0.54	0.70
CCT	P	1.19	1.14	1.02	1.13	1.14
CCC	P	0.42	0.52	0.55	0.48	0.61
CCA	P	0.76	0.69	0.75	0.74	0.50
CCG	P	**1.63**	**1.65**	**1.68**	**1.65**	**1.75**
ACT	T	0.99	1.19	1.23	1.17	1.19
ACC	T	**1.83**	**1.58**	**1.56**	**1.60**	**1.67**
ACA	T	0.24	0.25	0.25	0.28	0.26
ACG	T	0.94	0.98	0.96	0.95	0.89
GCT	A	**1.95**	**1.88**	**1.93**	**1.85**	**1.73**
GCC	A	0.66	0.65	0.64	0.73	0.66
GCA	A	0.61	0.68	0.71	0.58	0.68
GCG	A	0.77	0.78	0.72	0.84	0.93
TAT	Y	0.75	0.78	0.72	0.75	0.95
TAC	Y	**1.25**	**1.22**	**1.28**	**1.25**	**1.05**
CAT	H	**1.11**	**1.15**	**1.18**	**1.21**	**1.25**
CAC	H	0.89	0.85	0.82	0.79	0.75
CAA	Q	**1.09**	**1.14**	**1.18**	**1.24**	**1.28**
CAG	Q	0.91	0.86	0.82	0.76	0.72
AAT	N	**1.47**	**1.49**	**1.44**	**1.52**	**1.56**
AAC	N	0.53	0.51	0.56	0.48	0.44
AAA	K	0.02	0.07	0.08	0.03	0.02
AAG	K	**1.98**	**1.93**	**1.92**	**1.97**	**1.98**
GAT	D	**1.15**	**1.16**	**1.18**	**1.17**	**1.15**
GAC	D	0.85	0.84	0.82	0.83	0.85
GAA	E	**1.10**	**1.02**	**0.99**	**1.05**	**0.91**
GAG	E	0.90	0.98	1.01	0.95	1.09
TGT	C	0.00	0.02	0.00	0.00	0.02
TGC	C	**2.00**	**1.98**	**2.00**	**2.00**	**1.98**
CGT	R	1.07	1.01	1.00	1.04	1.06
CGC	R	0.03	0.01	0.01	0.02	0.01
CGA	R	**2.07**	**2.07**	**1.99**	**2.03**	**2.07**
CGG	R	0.00	0.03	0.04	0.00	0.01
AGA	R	1.00	0.86	0.92	0.96	0.50
AGG	R	1.83	2.03	2.04	1.95	2.34

GGT	G	**2.06**	**2.19**	**2.14**	**2.14**	**2.30**
GGC	G	0.00	0.00	0.01	0.00	0.01
GGA	G	0.84	0.76	0.77	0.78	0.69
GGG	G	1.10	1.06	1.08	1.09	1.00
