# Supplementary figures and images for: An Evolutionary Perspective of Codon Usage Pattern, Dinucleotide Composition and Codon Pair Bias in Prunus Necrotic Ringspot Virus
Source: Genes (Basel). 2023 Aug 28;14(9):1712. doi: 10.3390/genes14091712 (PMC10530913; doi:10.3390/genes14091712)

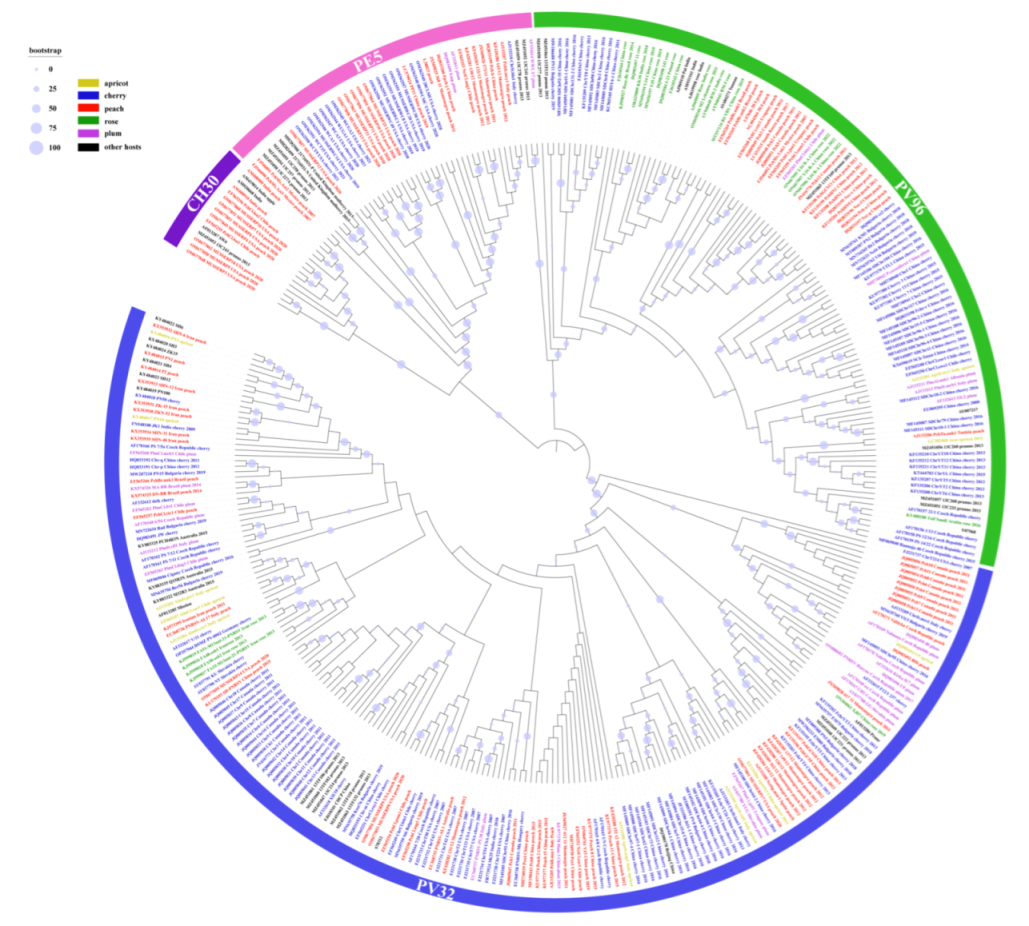

Supplement: Supplementary file 1 [file genes-14-01712-s001.zip › Figure S1.tif]

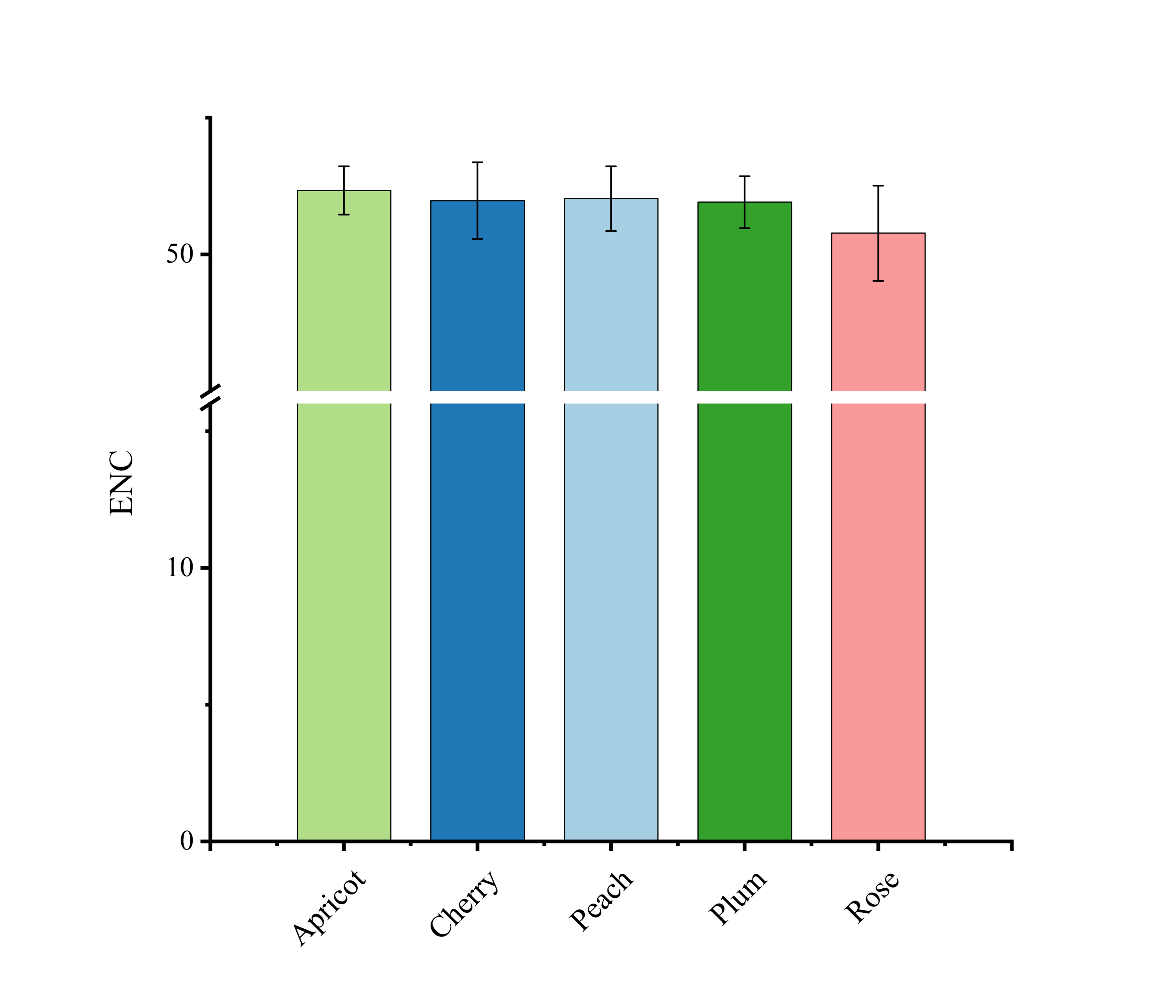

Supplement: Supplementary file 1 [file genes-14-01712-s001.zip › Figure S2.tif]

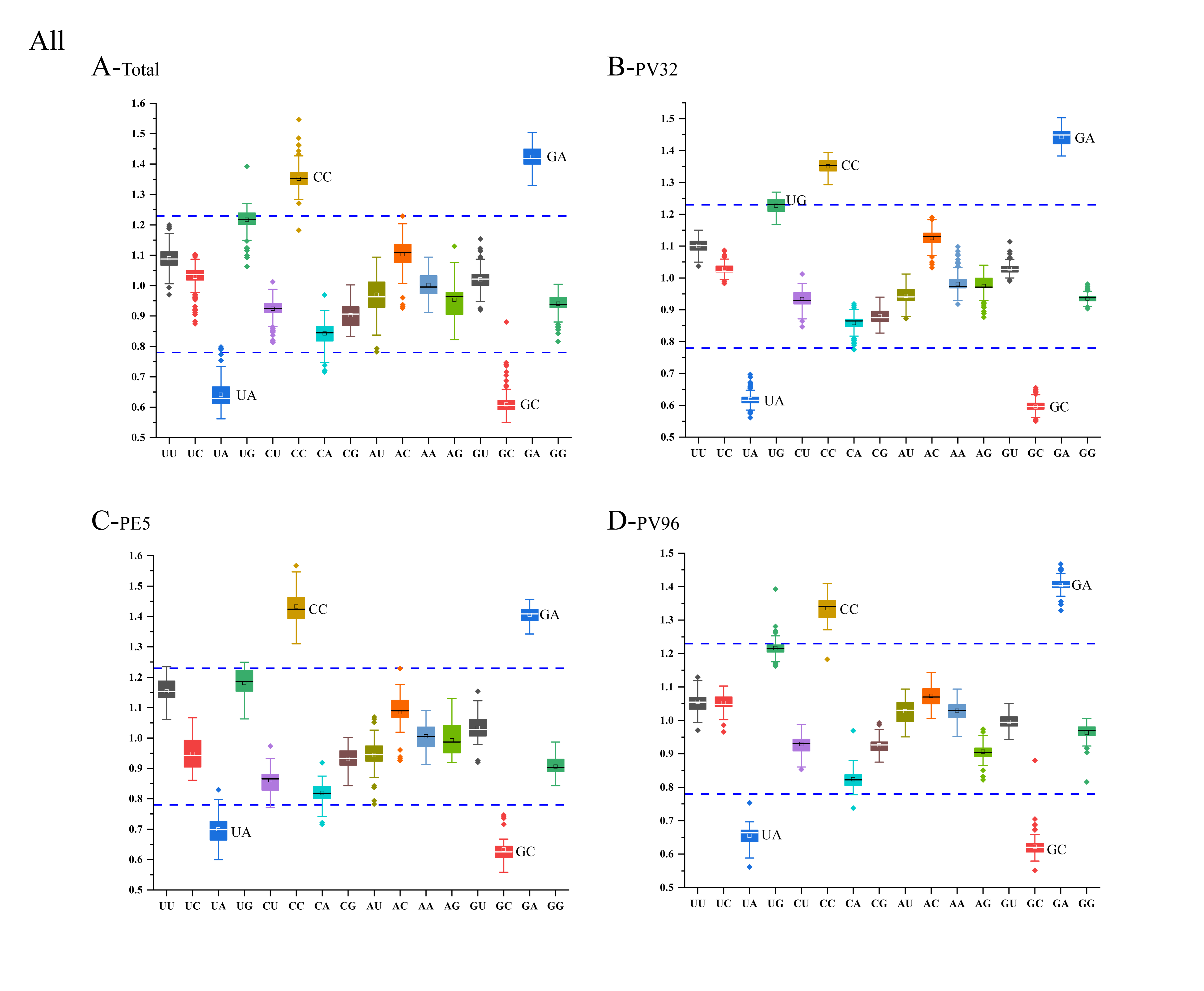

Supplement: Supplementary file 1 [file genes-14-01712-s001.zip › Figure S3.tif]

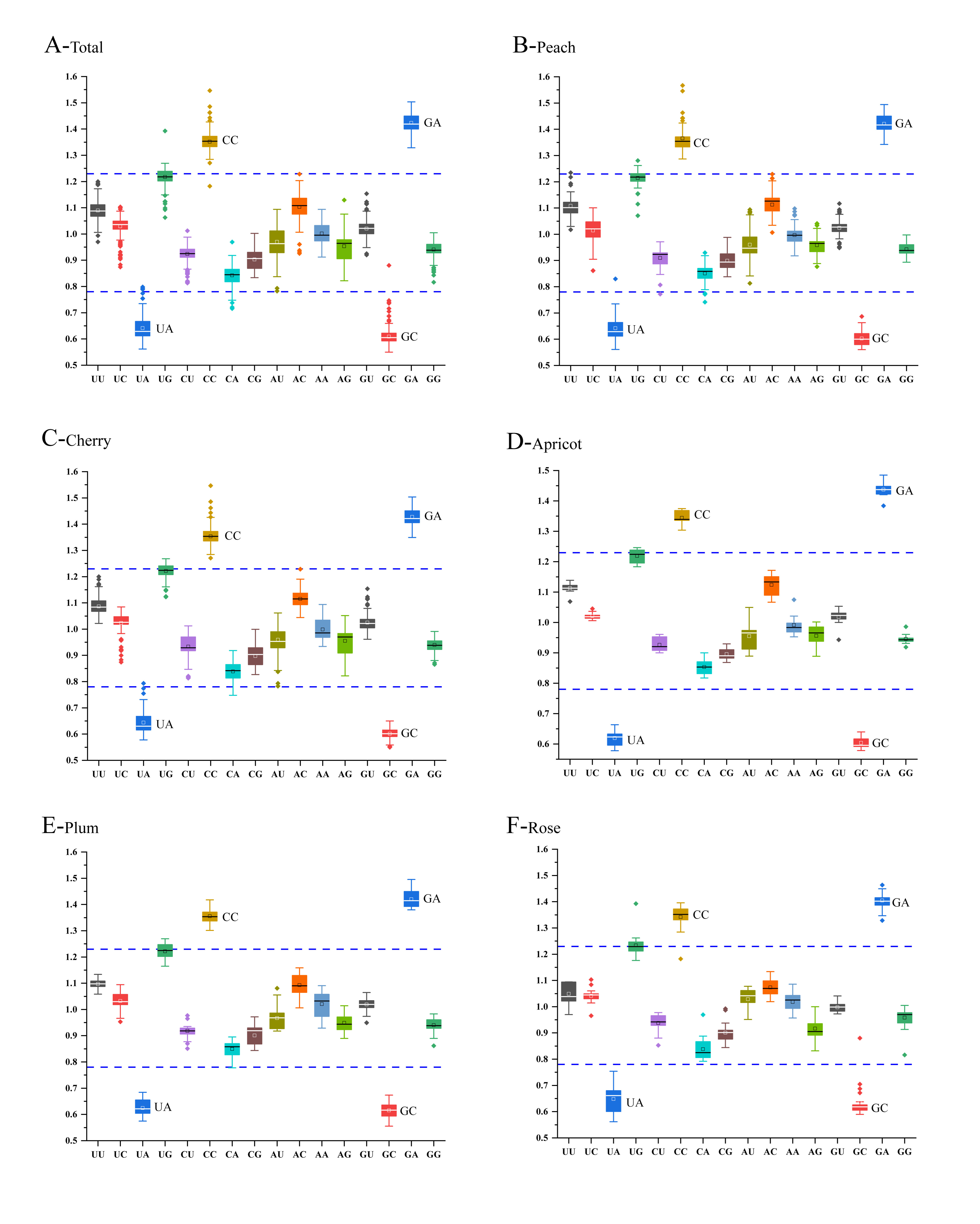

Supplement: Supplementary file 1 [file genes-14-01712-s001.zip › Figure S4.tif]

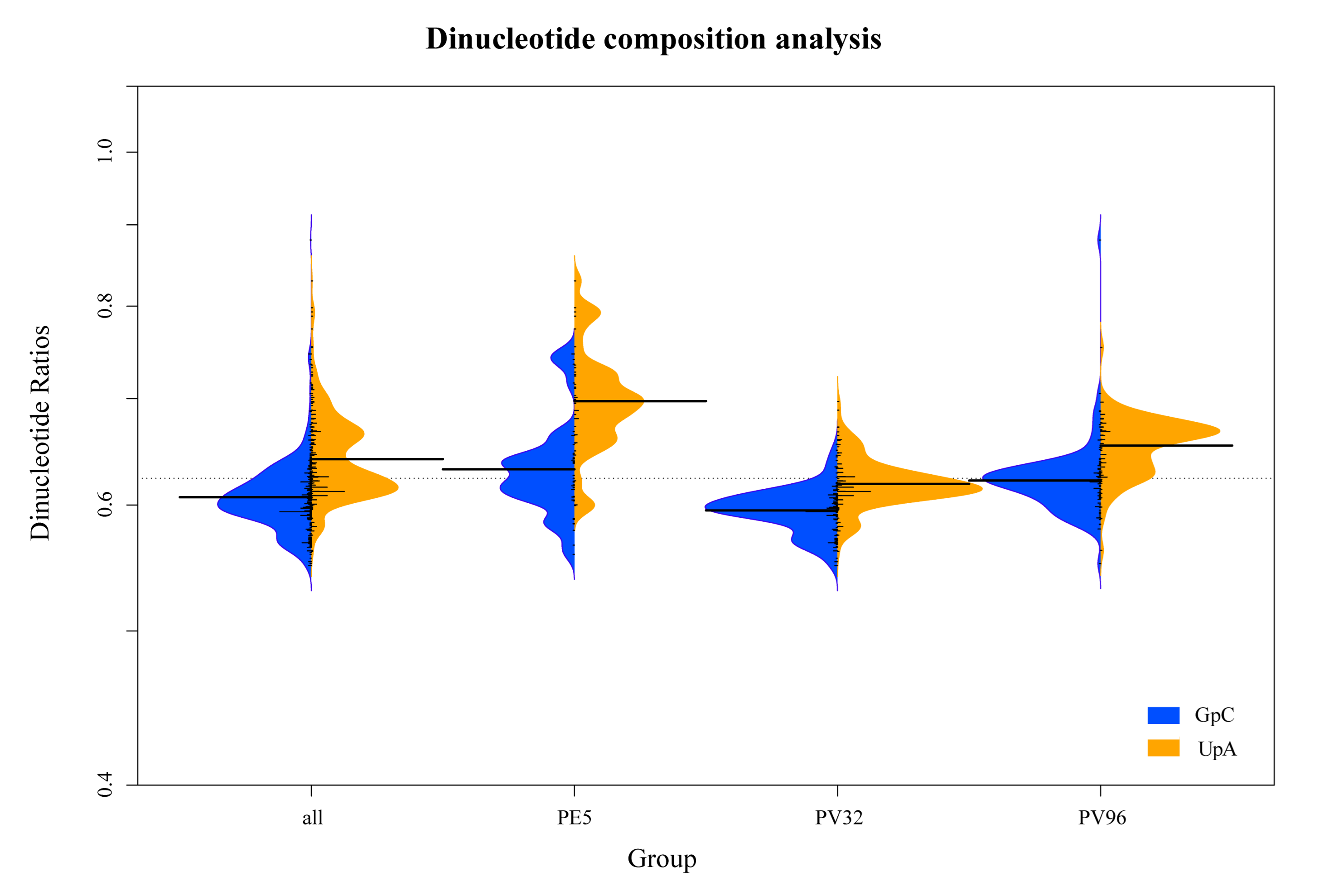

Supplement: Supplementary file 1 [file genes-14-01712-s001.zip › Figure S5.tif]

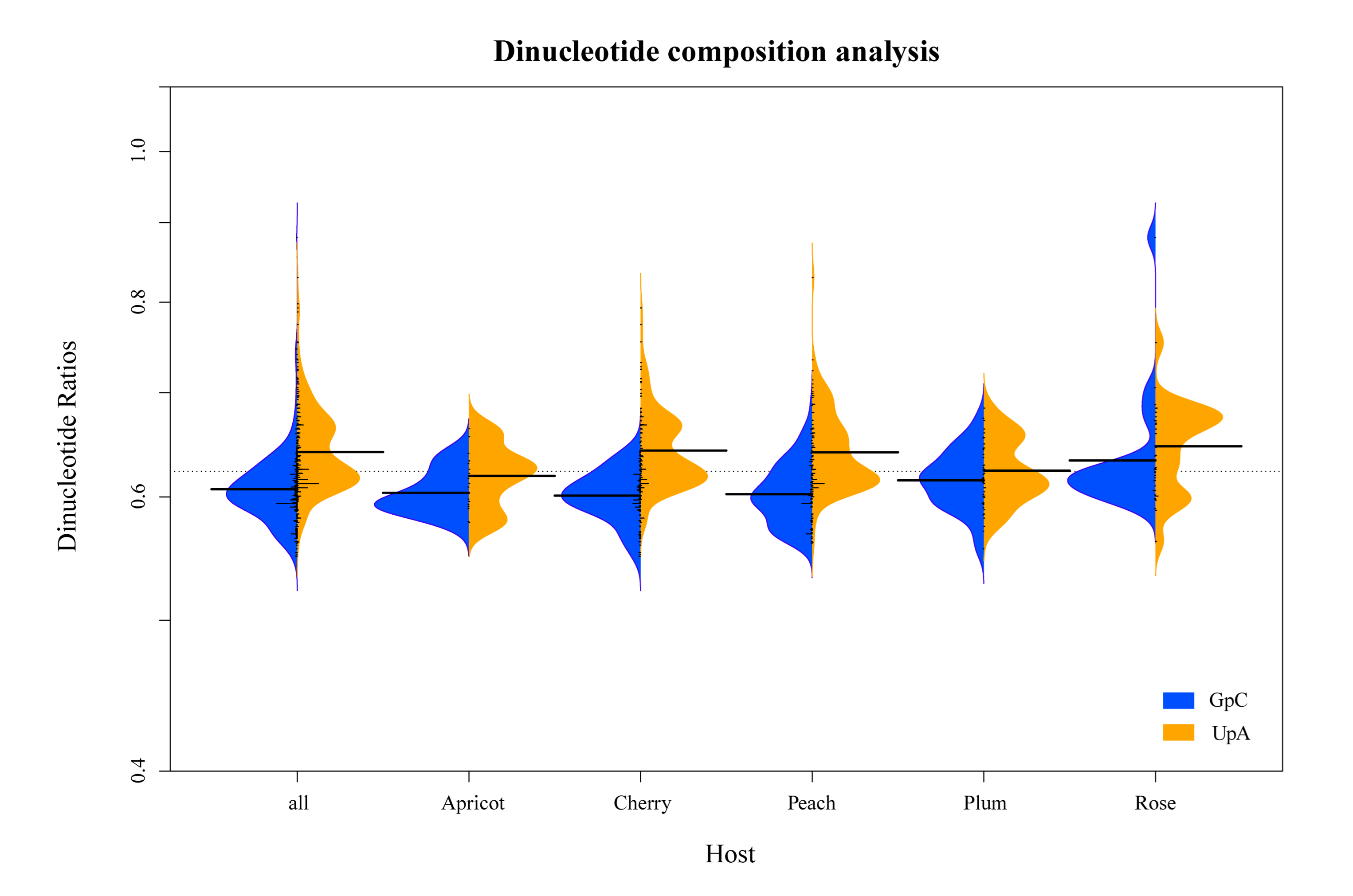

Supplement: Supplementary file 1 [file genes-14-01712-s001.zip › Figure S6.tif]
